# Evaluating the Association Between Educational Status and Carpal Tunnel Syndrome Presentations and Severity

**DOI:** 10.1155/aort/9975946

**Published:** 2025-08-10

**Authors:** Moh'd S. Dawod, Mohammad N. Alswerki, Ahmad F. Alelaumi, Taghleb Al-Awad, Abdualmajid Alameri, Abdulrahman Abu-Humdan, Nedal Alsabatin, Ala'a Altaher, Khaled Al-Amer, Aws Khanfar

**Affiliations:** ^1^Faculty of Medicine, Mutah University, Al-Karak, Jordan; ^2^Department of Orthopedic Surgery, Jordan University Hospital, Amman, Jordan; ^3^Orthopedic Department, Seiyun General Hospital, Sanaa, Yemen; ^4^Jordan University Hospital, University of Jordan, Amman, Jordan; ^5^Department of Orthopedic Surgery, Jordan University Hospital, University of Jordan, Amman, Jordan

**Keywords:** Boston Carpal Tunnel Questionnaire, carpal tunnel syndrome, educational status, hand pain

## Abstract

**Introduction:** Carpal tunnel syndrome (CTS), a painful prevalent orthopedic hand condition causing pain and paresthesia, is typically diagnosed clinically. Initial management involves analgesia trials, steroid injections, and night splints, with surgery as an option for failed conservative treatment. While prior research has explored the relationship between patients' educational status and various orthopedic conditions, no studies have investigated its association with clinical presentation and symptom severity in CTS. Therefore, our study aims to investigate this important link.

**Methods:** Our study utilized a retrospective study design, which included 681 patients undergoing carpal tunnel release surgery at a prominent teaching hospital. The aim was to investigate the association between four distinct educational levels and the clinical presentation and severity of the disease. Disease severity was evaluated using the Boston Carpal Tunnel Questionnaire (BCTQ).

**Results:** The study included individuals with a mean age of 52.0 years and diverse educational backgrounds: 20.0% high school, 34.9% diploma, 28.5% bachelor's degree, and 6.6% higher education qualifications. Subjective grip strength decline was more pronounced in high school and diploma categories (83.1% and 82.4%, respectively) compared to bachelor's and higher education categories (71.0% and 68.8%, respectively; *p*=0.005). Additionally, high school patients had higher Gabapentin usage for analgesia (32.4%) compared to other groups (*p*=0.014).

**Conclusion:** In patients with CTS, there is a correlation between lower education and symptoms of subjective weakened grip strength, increased analgesic use, and higher Gabapentin utilization. Conversely, higher education is associated with greater utilization of night splints. Moreover, postoperative improvements were observed across all educational groups with no significant differences.

**Level of Evidence:** Level III, Retrospective Study.

## 1. Introduction

Carpal tunnel syndrome (CTS) is a common peripheral neuropathic disorder caused by the compression of the median nerve as it passes through the confined carpal tunnel at the wrist [1]. It is characterized by pain, tingling, and numbness over the dermatomal area of the median nerve [2, 3]. In some cases, individuals with CTS may experience nocturnal symptoms and a reduction in grip strength [4, 5]. While the primary means of diagnosing CTS is through clinical assessment, electrodiagnostic studies can be employed to confirm the diagnosis [6, 7]. Surgical management is considered if conservative management proves ineffective [8].

Prior research has suggested that various determinants may influence the severity of symptoms and functional impairment observed in individuals diagnosed with CTS [9, 10]. Certain factors fall within the scope of demographic variables, such as age and gender [11]. Additional variables encompass aspects of the patient's health profile, including the presence of conditions such as diabetes and renal disease [12, 13]. Furthermore, the clinical presentation, specifically hand grip weakness, has been identified as a contributing factor to symptom severity [14]. It is notable that lower socioeconomic status is regarded as an indicator of suboptimal outcomes in this context [15].

Among these demographic and clinical variables, age, gender, and hand grip strength play a particularly important role in shaping symptom presentation. Increasing age has been associated with more pronounced symptom severity and delayed nerve conduction recovery [16]. Female patients tend to have a higher prevalence of CTS, possibly due to anatomical or hormonal differences [17]. Additionally, subjective hand grip weakness often reflects greater functional impairment and may indicate more advanced disease progression [18]. These factors collectively underscore the multifaceted nature of symptom severity in CTS.

Various orthopedic conditions, including knee osteoarthritis [19], upper extremity fractures [20], and back pain [21, 22], have demonstrated associations with educational status. Recognizing the mediating effect of educational status, it is important to explore its potential relationship with clinical presentation, symptom severity, and treatment-seeking behavior in CTS. Educational attainment may influence health literacy, access to care, awareness of treatment options, and occupational exposures—factors that could affect disease experience and outcomes. This is particularly important given the lack of comprehensive studies in literature specifically addressing this aspect.

Therefore, the aim of this study is to investigate the associations between the patient's educational status, clinical presentation, and severity of symptoms in patients with CTS.

## 2. Patients and Methods

The study implemented a retrospective design and was conducted at a major teaching hospital in Jordan over a timeframe of five years, spanning from January 2018 to December 2023. The patient group in our study comprised individuals who had undergone carpal tunnel release surgery within the defined period of the study.

The study's inclusion criteria encompassed patients who underwent isolated open carpal tunnel release surgery under local anesthesia within the specified study period. Exclusion criteria comprised individuals who underwent revision surgery, procedures under general anesthesia, combined interventions beyond carpal tunnel release, those lost to follow-up, patients with incomplete health records, and participants who declined study participation.

The data collected for this study encompassed various aspects, including demographic information such as age and gender, details on patients' medical comorbidities and overall health profiles, as well as information pertaining to clinical presentations, symptomatology, and the severity of symptoms.

The primary objective of this study was to examine the clinical presentations and symptom severity among individuals belonging to four specific educational categories. Data for this study were gathered through authorized access to patients' health records, in-person visits to clinics, and phone calls.

The educational status of the participants was categorized into four levels, ranging from the lowest to the highest: high school, diploma, bachelor's degree, and higher education (including master's and doctoral degrees). Data regarding the educational status of the patients were collected through self-reporting by the participants themselves.

To assess the severity of symptoms and derive meaningful insights from patients' reported symptomatology, we utilized the Boston Carpal Tunnel Questionnaire (BCTQ). This questionnaire was routinely administered and scored at the time of surgery in our hospital.

**Figure 1:**
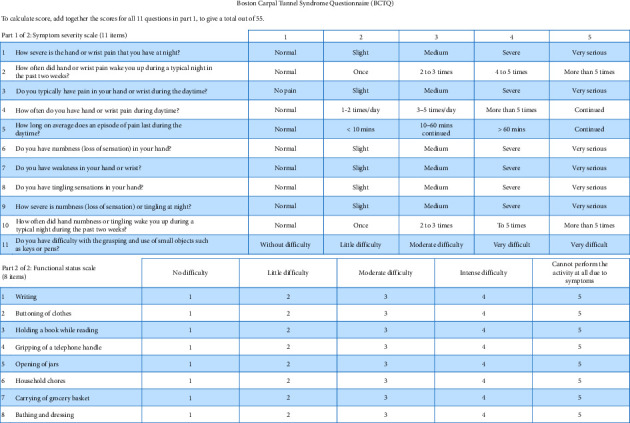
Boston Carpal Tunnel Questionnaire [24].

**Figure 2:**
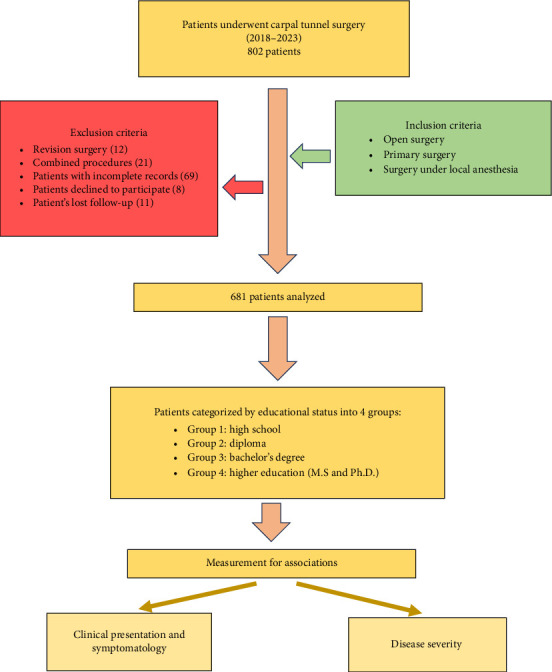
Flowchart summary of the study methodology.

The BCTQ, developed by Levine et al. in 1993 [23], consists of two distinct components. The first component comprises 11 questions and evaluates the severity of CTS symptoms. Each question is scored on a scale of 1–5, with lower scores indicating better conditions (1 being the best and 5 being the worst). The second component assesses the functional status of patients using eight items, with each item also scored on a scale of 1–5. Again, lower scores indicate more favorable conditions (1 being the best and 5 being the worst). Upon completion of the questionnaire, the mean values for both the symptom and functional scales are calculated to provide an overall assessment of patients' symptom severity and functional impairment.  Figure 1 shows the BCTQ.  Figure 2 presents a flowchart that summarizes the study's methodology.

Moreover, follow-up BCTQ scores were obtained either during patients' clinic visits or through phone calls. The differences between the preoperative scores (initially documented at the time of surgery) and the postoperative scores were calculated and compared across the four educational groups. This analysis aimed to provide insights into the clinical improvement experienced by patients in each of the four studied groups.

An appropriate institutional review board was obtained prior to the conduction of this study from our hospital's ethics committee, IRB approval number (2023/25128). Appropriate informed consents were obtained from all participants of the study. The Code of Ethics of the World Medical Association (Declaration of Helsinki) was followed while conducting the study. Data were recorded and analyzed using the Statistical Package for Social Science (SPSS), version 23.

Data were recorded and analyzed using SPSS Version 23. A priori power analysis was conducted (with parameters set at *α* = 0.05 and power = 0.80) to ensure that our sample size was adequate for detecting significant differences. Descriptive statistics were generated to summarize continuous variables (reported as means ± standard deviations) and categorical variables (expressed as frequencies and percentages). For continuous outcomes, such as age and pre-/postoperative BCTQ scores, independent samples *t*-tests were employed for two-group comparisons, while one-way analysis of variance (ANOVA) was used for comparisons across more than two groups. For categorical data—including gender, occupation, and treatment modalities—Pearson's chi-square test was applied, with Fisher's exact test utilized when expected cell counts were below five. A two-tailed *p* value of less than 0.05 was considered statistically significant.

This comprehensive statistical framework ensured a rigorous evaluation of the association between educational status and various clinical outcomes in CTS, effectively capturing central tendencies and distributional differences while minimizing potential confounding effects.

## 3. Results

The study encompassed an analysis of 681 patients, with a mean age of 52.0 years. Among these, 526 patients (77.2%) were females and 155 patients (22.8%) were males. Surgical interventions were performed on the right hand for 421 patients (61.8%), while 260 patients (28.2%) underwent surgery on the left hand. The majority of the cohorts, constituting 92.7%, were right-handed individuals.

In terms of educational status, a total of 136 patients (20.0%) had completed high school, 238 patients (34.9%) had obtained a diploma, 262 patients (28.5%) held a bachelor's degree, and 24 patients (6.6%) had pursued higher education (Table 1).

The comparative analysis of clinical presentations across educational categories revealed consistent trends. Regardless of educational status, a significant proportion of patients reported significant daytime pain, with figures ranging from 83.2% to 92.6%. Similarly, paresthesia was very common across all educational groups, with percentages ranging from 94.7% to 100.0%. The statistical analysis did not show significant differences between educational groups for both daytime pain (*p*=0.06) and numbness/paresthesia (*p*=0.23) (Table 2).

Night symptoms as a presentation showed a similar pattern across all educational categories, with percentages ranging from 81.6% to 91.1%. However, concerning decreased grip strength, patients within the high school and diploma categories displayed higher percentages (83.1% and 82.4%) compared to those in the master's and doctoral categories (71.0% and 68.8%, respectively; *p*=0.005) (Table 2).

Moreover, individuals with higher education levels had a higher prevalence of reporting additional musculoskeletal symptoms such as neck pain (44.4%) and shoulder pain (51.1%). In contrast, those with lower educational levels exhibited lower rates of reporting such symptoms. These findings suggest a potential correlation between higher educational attainment and the reporting of musculoskeletal symptoms (Table 2).

Lower educational attainment levels were inferentially associated with increased analgesic usage across educational groups. Specifically, individuals with high school diplomas and diplomas reported higher rates of analgesic use compared to those with bachelor's and higher education degrees (*p*=0.002). Additionally, patients in the high school category had a higher utilization of Gabapentin for analgesia (32.4%) compared to the diploma (26.5%), bachelor (18.3%), and higher education (26.7%) groups (*p*=0.014) (Table 3).

However, no statistically significant differences were observed across the four educational categories in terms of the use of steroid injections (*p*=0.40), utilization of night splints (*p*=0.63), receiving adequate preoperative counseling (*p*=0.88), and experiencing excessive anxiety (*p*=0.74) (Table 3).

When comparing postoperative outcomes and recovery among different educational groups, the findings revealed the following results. Using the BCTQ scoring system to evaluate differences in symptom severity (S score in Table 4), the mean differences were nearly the same across all four categories, indicating similar improvements in symptom severity. Similarly, when examining the differences in functional scores (F score in Table 4), there were closely related mean differences across the four educational groups, suggesting comparable functional improvement after surgery in all four categories (Table 4).

## 4. Discussion

Our study compared various educational levels with CTS presentations and severity. Demographically, our cohort across all educational groups was predominantly female and composed largely of nonsmokers. These findings are consistent with existing literature. Multiple studies have shown that CTS is more prevalent in females, which may be attributed to anatomical differences such as smaller carpal tunnel dimensions, hormonal influences, and occupational roles involving repetitive hand use [25, 26].

In terms of smoking, our cohort demonstrated relatively low rates across all educational groups, with no significant associations observed between smoking status and clinical presentations, treatment modalities, or postoperative outcomes. This aligns with previous research where the role of smoking as a risk factor for CTS remains inconclusive; while some studies suggest a modest association, others report no clear link with symptom severity or surgical prognosis [3, 27, 28].

Comorbidities such as hypertension and diabetes were common among our patients, aligning with established risk profiles in CTS populations [29, 30]. Furthermore, nearly all patients across all groups reported core symptoms including daytime pain, paresthesia, and nocturnal disturbances—hallmark features described in most CTS clinical series [31, 32].

Patient educational status has been linked to various healthcare outcomes across orthopedic conditions. For example, Olson et al. found that higher educational attainment was associated with significantly greater improvement in nonoperative treatment for lumbar disc herniation, although it did not influence surgical outcomes [33]. Similarly, Paksima et al. demonstrated that higher education levels are associated with improvements in pain, range of motion, grip strength, and disability scores after distal radius fractures [20]. Furthermore, Moulton et al. reported that preoperative education before hip arthroplasty significantly reduces hospital stay length and costs while positively impacting mobilization and outcome scores [34]. These findings underscore that educational status is an important factor to investigate in orthopedic conditions.

In our study, an association was found between CTS patients with lower educational status and subjective weakened grip strength, regular analgesic use, and receiving Gabapentin compared to those with higher educational attainment. This observation suggests potential underlying mechanisms. Individuals with lower educational levels are often employed in manual labor occupations, which can contribute to CTS or worsen symptoms due to repetitive movements and strain. Additionally, socioeconomic challenges faced by those with lower educational status, such as limited access to healthcare and economic pressures to continue working despite symptoms, may result in delayed presentation until symptoms reach intolerable levels. These factors collectively underscore the complex interplay between educational status, occupational factors, socioeconomic status, and symptom severity in CTS patients.

While socioeconomic status (SES) is undoubtedly intertwined with educational attainment, our study deliberately focused on education as a primary, quantifiable marker of health literacy and patient preferences. SES, encompassing factors such as income, occupation, and access to healthcare, could potentially influence both the presentation and management of CTS. However, by not directly measuring SES, we aimed to maintain methodological clarity and consistency, avoiding the additional complexity and potential multicollinearity that may arise from incorporating multiple interrelated variables. Although occupation was used as a proxy to capture some aspects of socioeconomic standing, this measure does not fully encapsulate the broader socioeconomic environment, including income levels, neighborhood characteristics, and insurance status.

We recognize that lower SES is often associated with reduced access to healthcare services, delayed treatment, and limited availability of conservative management options, factors that may further exacerbate CTS symptoms. This limitation underscores the need for future research to adopt a more comprehensive approach by directly assessing SES alongside educational status. Prospective, multicenter studies incorporating validated socioeconomic indices could provide deeper insights into how these factors interact to influence CTS severity and treatment outcomes, ultimately guiding more targeted and equitable health policy interventions. In summary, while our findings highlight the impact of educational status on CTS management and patient preferences, the exclusion of a direct SES assessment remains a methodological limitation that warrants further investigation.

Furthermore, the cohort of higher education patients demonstrated increased utilization of night splints as an adjunct for pain management in comparison to other groups. This finding could be potentially elucidated by two factors. Firstly, individuals with higher educational status may be more inclined to seek information about their condition, treatment modalities, and available interventions compared to those with lower educational attainment. Secondly, this subset of patients exhibited more nocturnal symptoms in contrast to the other groups, providing an additional explanation for our observed result.

Previous literature has associated various factors with the clinical presentation and symptomatology of CTS. Daliri et al. linked psychological distress, including depression and anxiety, to increased hand disability in CTS patients [35]. Maghsoudipour et al. identified occupational factors such as force exertion, bending/twisting of hands, rapid hand movement, and vibration as linked to CTS [36]. Sharief et al. reported that female gender, diabetes mellitus, hypertension, and impaired thyroid functions can precipitate CTS [30]. Kaplan et al. emphasized the significance of older age and a longer duration of symptoms in predicting symptom severity [37]. Notably, to the best of the authors' knowledge, no prior literature has investigated the educational status of CTS patients and its effect on clinical presentation and symptom severity.

In close context, in their study published in 2025, Göktürk and colleagues evaluated the Beck depression scale scores of 100 patients diagnosed with CTS and showed that although it was relatively low among university graduates, it was significantly higher in those who described their economic status as poor, lived in rural areas, and were unemployed [38].

The BCTQ is recognized as a reliable tool for objectively assessing disease severity in CTS patients. Leite et al. [39] and subsequent studies on language-specific versions, including Chinese [40], Polish [41], and Greek [42], confirm its validity, reliability, and responsiveness. The BCTQ's versatility across languages underlines its effectiveness in providing accurate and objective assessments of symptom severity in diverse cultural contexts, making it a valuable tool for both clinical and research purposes. In our study, we utilized the BCTQ to obtain assessments of disease severity from the patients' perspectives, allowing for a comprehensive understanding of the impact of CTS.

While we acknowledge certain limitations in our study, it is argued that these constraints do not undermine the validity of our findings. The retrospective design, despite potentially introducing limitations in data collection and analysis, was invaluable for exploring the observed associations. However, the single-center setting may limit generalizability, though our institution serves a diverse patient population. Future multicenter studies are needed to validate these findings. Despite the modest size of our patient sample, the data extracted from this cohort retains considerable value. Our analysis focused on educational status, which may not fully account for the influence of socioeconomic status and healthcare accessibility.

While we did not directly assess socioeconomic status, we included occupation as a proxy due to its correlation with both education and economic standing. This approach ensured methodological clarity while acknowledging the complex interplay between these factors. Future studies incorporating comprehensive socioeconomic metrics could further elucidate their role in CTS severity and treatment patterns.

## 5. Conclusion

Lower educational status was associated with lower grip strength, increased use of analgesic modalities, and a higher frequency of Gabapentin use. On the other hand, individuals with higher educational status were more likely to report additional musculoskeletal symptoms related to CTS and showed increased use of night splints. It is worth noting that postoperative symptomatic and functional improvements were consistently observed across all four educational groups.

### 5.1. Contributions to Literature

1. This study offers valuable insights into how educational status impacts the presentation and treatment of a common condition, CTS, highlighting the role of health disparities in patient care approaches and management, particularly within health and orthopedic practice.
2. The findings advocate for enhanced health education and promotion strategies that address gaps in knowledge, empowering patients with lower educational attainment to seek timely care and adhere to treatment.
3. By integrating educational status into health system planning, this research promotes health equity and supports public health initiatives aimed at reducing socioeconomic barriers to care.
4. This study highlights the importance of targeted health interventions and patient-centered communication strategies to improve health literacy, ensure equitable access to treatment, and optimize health outcomes across diverse populations.

## Figures and Tables

**Table 1:** Distribution of medical comorbidities according to the educational status category of the patients.

Comparison health profiles		High school (*n* = 136) *n* (%)	Diploma (*n* = 238) *n* (%)	Bachelor's degree (*n* = 262) *n* (%)	Higher education (*n* = 45) *n* (%)
Gender	Male	21 (15.4%)	48 (20.2%)	66 (25.2%)	20 (44.4%)
	Female	115 (84.6%)	190 (79.8%)	196 (74.8%)	25 (55.6%)

Occupation	Manual	87 (64.0%)	140 (58.8%)	72 (27.5%)	3 (6.0%)
	Nonmanual	49 (36.0%)	98 (41.2%)	190 (72.5%)	42 (94.0%)

Smoking status	Smokers	16 (11.8%)	51 (21.4%)	48 (18.3%)	7 (15.6%)
	Nonsmokers	120 (88.2%)	187 (78.6%)	214 (81.7%)	38 (84.4%)

Hypertension	Yes	69 (50.7%)	95 (39.9%)	85 (32.4%)	13 (28.9%)
	No	67 (49.3%)	143 (60.1%)	177 (67.6%)	32 (71.1%)

Diabetes	Yes	67 (49.3%)	69 (29.0%)	71 (27.1%)	12 (26.7%)
	No	69 (50.7%)	169 (71.0%)	191 (72.9%)	33 (73.3%)

Cardiovascular disease	Yes	37 (27.2%)	34 (14.3%)	17 (6.5%)	5 (11.1%)
	No	99 (72.8%)	204 (85.7%)	245 (95.5%)	40 (88.9%)

Cerebrovascular disease	Yes	14 (10.3%)	26 (10.9%)	19 (7.3%)	6 (13.3%)
	No	122 (89.7%)	212 (89.1%)	243 (92.7%)	39 (86.7%)

Renal disease	Yes	7 (5.1%)	8 (3.4%)	8 (3.1%)	1 (2.2%)
	No	129 (94.9%)	230 (96.6%)	254 (96.9%)	44 (97.8%)

Rheumatological disease	Yes	18 (13.2%)	21 (8.8%)	20 (7.6%)	4 (8.9%)
	No	118 (86.8%)	217 (91.2%)	242 (92.4%)	41 (91.1%)

**Table 2:** Comparison between clinical presentations across the four educational groups.

Comparison clinical presentation		High school (*n* = 136) *n* (%)	Diploma (*n* = 238) *n* (%)	Bachelor's degree (*n* = 262) *n* (%)	Higher education (*n* = 45) *n* (%)	Chi-square (*p* value)
Daytime pain	Yes	126 (92.6%)	207 (87.0%)	218 (83.2%)	39 (86.7%)	7.18 (0.06)
	No	10 (7.4%)	31 (13.0%)	44 (16.8%)	6 (13.3%)	

Paresthesia^a^	Yes	133 (97.8%)	228 (95.8%)	248 (94.7%)	45 (100.0%)	4.30 (0.23)
	No	3 (2.2%)	10 (4.2%)	14 (5.3%)	0 (0.0%)	

Night pain^b^	Yes	111 (81.6%)	208 (87.4%)	235 (89.7%)	41 (91.1%)	5.99 (0.11)
	No	25 (18.4%)	30 (12.6%)	27 (10.3%)	4 (8.9%)	

Weak grip^c^	Yes	113 (83.1%)	196 (82.4%)	186 (71.0%)	31 (68.8%)	12.6 **(0.005)**^**∗**^
	No	23 (16.9%)	42 (17.6%)	76 (29.0%)	14 (31.1%)	

Neck pain	Yes	41 (30.1%)	73 (30.7%)	82 (31.3%)	20 (44.4%)	3.66 (0.33)
	No	95 (69.9%)	165 (69.3%)	180 (68.7%)	25 (55.6%)	

Shoulder pain	Yes	63 (46.3%)	86 (36.1%)	102 (38.9%)	23 (51.1%)	6.16 (0.10)
	No	73 (53.7%)	152 (63.9%)	160 (61.1%)	22 (48.9%)	

Elbow pain	Yes	65 (47.8%)	98 (41.2%)	128 (48.9%)	22 (48.9%)	3.73 (0.27)
	No	71 (52.2%)	140 (58.8%)	134 (51.1%)	23 (51.1%)	

*Note:* Bolded values denoted by an asterisk indicate statistically significant values (*p* < 0.05).

^a^Paresthesia across the medial three fingers at the distribution of the median nerve.

^b^Night pain that is significant enough to awaken patients from sleep or cause significant distress, with or without paresthesia.

^c^Subjective patient-reported feeling of weakened hand grip interfering with daily activities.

**Table 3:** Comparison of treatment modalities received across the four educational categories.

Comparison treatment received		High school (*n* = 136) *n* (%)	Diploma (*n* = 238) *n* (%)	Bachelor's degree (*n* = 262) *n* (%)	Higher education (*n* = 45) *n* (%)	Chi-square (*p* value)
Regular analgesia use^a^	Yes	101 (74.3%)	148 (62.2%)	149 (56.9%)	22 (48.9%)	14.8 **(0.002)**^**∗**^
	No	35 (25.7%)	90 (37.8%)	113 (43.1%)	23 (51.1%)	

Injections^b^	Yes	25 (18.4%)	36 (15.1%)	33 (12.6%)	5 (11.1%)	2.9 (0.40)
	No	111 (81.6%)	202 (84.9%)	229 (87.4%)	40 (88.9%)	

Night splints use^c^	Yes	44 (32.4%)	86 (36.1%)	89 (34.0%)	19 (42.2%)	1.7 (0.63)
	No	92 (67.6%)	152 (63.9%)	173 (66.0%)	26 (57.8%)	

Gabapentin use^d^	Yes	44 (32.4%)	63 (26.5%)	48 (18.3%)	12 (26.7%)	10.5 **(0.014)**^**∗**^
	No	92 (67.6%)	175 (73.5%)	214 (81.7%)	33 (73.3%)	

Preoperative counseling^e^	Yes	120 (88.2%)	203 (85.3%)	227 (86.6%)	39 (86.7%)	0.65 (0.88)
	No	16 (11.8%)	35 (14.7%)	35 (13.4%)	6 (13.3%)	

Procedural experience^f^	Comfortable	95 (69.6%)	158 (66.4%)	171 (65.3%)	32 (71.1%)	1.2 (0.74)
	Anxious	41 (30.1%)	80 (33.6%)	91 (34.7%)	13 (28.9%)	

*Note:* Bolded values denoted by an asterisk indicate statistically significant values (*p* < 0.05).

^a^Regular analgesic use is defined as the daily consistent intake of analgesic medications for at least 1 week to alleviate pain caused by carpal tunnel syndrome.

^b^Corticosteroid injections for the treatment of carpal tunnel syndrome.

^c^Use of a night splint by patients with carpal tunnel syndrome purposefully to relieve nocturnal symptoms.

^d^Gabapentin is used specifically for the treatment of hand paresthesia associated with carpal tunnel syndrome, and not for other neuropathies.

^e^Counseling that adequately addresses patient concerns, perioperative care, treatment options, benefits, and risks of proposed surgery, and is well received by patients.

^f^Assessment of patient comfort levels during the procedure, including feelings of comfort or exaggerated anxiety/irritability.

**Table 4:** Comparison of the disease impact and postoperative recovery among the four educational groups.

Comparison disease impaction and recovery (means)	High school (*n* = 136) *n* (%)	Diploma (*n* = 238) *n* (%)	Bachelor's degree (*n* = 262) *n* (%)	Higher education (*n* = 45) *n* (%)	*p* value
Pre-op total *S* score	3.23	3.20	3.16	3.26	0.72
Post-op total *S* score	1.53	1.45	1.45	1.30	0.64
*S* score differences	−1.69	−1.74	−1.69	−1.95	0.39
Pre-op total *F* score	2.86	2.70	2.65	2.69	0.57
Post-op total *F* score	1.52	1.43	1.40	1.15	0.44
*F* score differences	−1.33	−1.26	−1.24	−1.54	0.20

## Data Availability

The data that support the findings of this study are available upon appropriate request from the corresponding author. The data is not publicly available due to privacy or ethical restrictions.

## Nomenclature

CTS: Carpal tunnel syndrome
BCTQ: Boston Carpal Tunnel Questionnaire
